# Analysis of key performance indicators influencing offensive effectiveness during clutch moments in elite international men’s basketball

**DOI:** 10.5114/biolsport.2026.163367

**Published:** 2026-07-27

**Authors:** Zhengyang Chen, Mingjia Qiu, Pierpaolo Sansone, Miguel-Ángel Gómez, Mingxin Zhang

**Affiliations:** 1 School of Athletic Performance, Shanghai University of Sport, Shanghai, China; 2 Shanghai Key Lab of Human Performance (Shanghai University of Sport), Shanghai, China; 3 Department of Education and Sport Sciences, Pegaso Telematic University, Naples, Italy; 4 Faculty of Physical Activity and Sport Sciences. Universidad Politécnica de Madrid, Madrid, Spain

**Keywords:** FIBA Basketball World Cup, Clutch time, Performance analysis, Notational analysis, Machine learning

## Abstract

Clutch moments often determine the final outcome of basketball games. The present study proposes an innovative analytical framework that integrates machine learning with explainable techniques to identify and quantify the influence of technical–tactical indicators on possession-level scoring outcomes during clutch moments. Using an observational methodology, 607 clutch offensive possessions from 41 games of the 2019 and 2023 FIBA Men’s Basketball World Cups were coded and analyzed. First, chi-square tests were applied to examine associations between technical–tactical variables and offensive outcomes (scoring vs. non-scoring). Subsequently, ten categories of machine learning models were systematically compared, with the Random Forest identified as the best-performing model for predicting scoring outcomes in clutch possessions. Finally, SHAP values were used to interpret the contribution and directional effects of each variable on model outputs. The results indicated that play-types (p < 0.001), possession duration (p = 0.009), and ending zone (p = 0.001) were significantly associated with scoring success. SHAP analysis further showed that “restricted area” “possessions completed within 1–8 seconds,” and “ending player position_center” were positively associated with scoring success. These findings provide data-driven support and practical reference for coaches and performance analysts when designing training priorities and tactical strategies in clutch contexts. Specifically, during clutch moments of elite international competition, offenses may benefit from selectively increasing tempo to break deadlocks and emphasizing team-oriented actions by increasing center involvement as primary finishers and strengthening screening and pick-and-roll actions.

## INTRODUCTION

Basketball is a team sport characterized by both confrontation and cooperation. In recent years, researchers have increasingly recognized the importance of distinguishing between two complementary frameworks for evaluating basketball performance: static game-related statistics derived from box-score data and dynamic, possession-level behavioral analysis [1, 2]. Traditional key performance indicators (KPIs), largely obtained from post-game statistical records, provide relatively comprehensive and objective summaries of players’ and teams’ contributions. Evidence has shown that match outcomes are significantly associated with indicators such as two-point and three-point shooting accuracy, free-throw accuracy, offensive rebounds, assists, and game pace [2, 3]. Moreover, assists, steals, and blocks have been used to differentiate high- from low-performing players [1, 4]. Despite the value of these game-related statistics in capturing overall performance contributions, they are inherently aggregated and descriptive at the game or player level, thereby limiting mechanistic insight into how specific within-possession decisions and interaction patterns translate into scoring advantages. This limitation may be particularly pronounced in high-pressure situations such as late-game phases, where decision-making constraints and tactical choices can differ substantially. To overcome these limitations, recent studies have increasingly adopted possession-level analytical frameworks to examine the dynamic structure of basketball performance [5, 6]. Such frameworks can quantify the frequency, duration, and sequential structure of events within each possession, generating datasets suitable for temporal or sequential analyses and facilitating the identification of offensive patterns and their underlying formation mechanisms [7, 8].

Observational methods are used to systematically capture technical–tactical behaviors and contextual dynamics within real-game environments [9]. Beyond possession-based analyses, observational methods can also be applied to individual behaviors, team interaction patterns, and spatiotemporal structures. In basketball research, observational methods have become increasingly valuable because they enable researchers to quantify technical–tactical behaviors and contextual constraints that cannot be adequately captured through aggregated performance indicators alone [5, 6].

Dynamic analysis of clutch moments is crucial because performance during these phases often has a decisive influence on game outcomes [10]. However, previous studies investigating clutch performance in basketball still exhibit several limitations. The existing research has primarily relied on static box-score statistics as analytical variables, making it difficult to capture the underlying technical–tactical processes and contextual interaction mechanisms associated with clutch performance [11–13]. More recently, some studies have adopted possession-based analytical approaches to provide a more detailed description of game behaviors. Nevertheless, these investigations have generally focused on specific scenarios, such as pick-and-roll possessions or after-timeout offensive possessions, while the categories of analyzed variables have remained insufficiently comprehensive [14, 15]. The literature still lacks an integrative framework within a unified international competitive setting that combines multidimensional technical, tactical, and contextual indicators to systematically quantify and compare clutch offensive performance. Therefore, further investigation of offensive behaviors during clutch phases in elite international tournaments and the identification of key determinants of offensive success is of both scientific and practical significance.

Existing research has shown that, compared with traditional scoring statistics, play-types during possession-level better reflect tactical decision-making on the court [10, 16, 17]. As a meso-level structure linking tactical intention, possession process, and scoring outcome, play-types can more directly capture teams’ tactical preferences and their adaptation to competitive environments, thereby providing a more interpretable perspective for understanding decision-making mechanisms at the possession level. Moreover, systematic differences in the use and efficiency of play-types across leagues have been documented, suggesting that conclusions regarding tactical effectiveness are highly context-dependent, such as league ecology, physical intensity, and rule environments [10]. Beyond play-types, the dynamic characteristics of possessions can be described through several key dimensions, including spatiotemporal constraints, players’ roles/positioning and numerical configurations during tactical execution, passing and interactive tactical behaviors, and spatial attributes such as shooting zones [6, 8]. Collectively, these dimensions provide a comprehensive representation of possession behaviors and constraints, forming the fundamental basis for possession-level dynamic analysis and for interpreting variations in offensive outcomes. In particular, timeouts during clutch moments represent an important contextual factor influencing offensive performance. In fact, previous research has indicated that calling a timeout may actually reduce the probability of successful scoring by 5.7% [18].

Our conceptual framework posits that, during clutch moments, offensive possession outcomes are not determined by a single factor but arise from the joint influence of contextual constraints, tactical choices, and finishing quality. Specifically, contextual factors such as score margin, possession-start situations, and whether the possession follows a timeout are assumed to shape teams’ tactical selections and organizational processes within the possession (e.g., play-types, number of passes, and the finisher’s role), which in turn influence proximal indicators of finishing quality, including shot timing (possession duration) and finishing zone, ultimately differentiating scoring from non-scoring possessions [8, 10, 16]. Based on this framework, we expect finishing zone and possession duration to be the most proximal determinants of scoring success, and that both the selection tendencies and success probabilities will differ across play-types and finisher roles in clutch moments.

Offensive performance during clutch moments is inherently multidimensional and dynamic, emerging from the complex interaction among contextual constraints, tactical choices, temporal characteristics, spatial organization, and player involvement. Under such conditions, traditional statistical techniques may be limited in their ability to adequately capture non-linear relationships, higher-order interactions, and complex dependency structures among variables [19]. Compared with conventional statistical approaches, machine-learning models are capable of simultaneously processing large numbers of interrelated variables without relying on strict parametric assumptions, while also providing superior capacity for modeling non-linear patterns and interaction effects [20]. This is particularly important in clutch moments, where offensive success may not result from isolated factors, but rather from the combined influence of tactical execution, possession duration, play-type selection, spatial finishing context, and situational constraints [21].

At present, systematic research on offensive performance during clutch moments in elite men’s basketball remains relatively limited, particularly with respect to identifying the key factors influencing possession-level scoring outcomes. To address this gap, the present study integrates observational analysis with machine learning–based modeling approaches, aiming to: (1) identify the key technical–tactical contextual factors associated with offensive success during clutch moments in elite men’s basketball; (2) compare the predictive performance of ten different categories of machine learning models to determine the optimal model; and (3) employ SHAP (SHapley Additive exPlanations) analysis to quantify the contribution and directional effects of each variable on model outputs, thereby enhancing model interpretability and supporting practical applications.

## MATERIALS AND METHODS

### Design and participants

This study employed an observational research methodology to achieve its stated objectives [9]. A multidimensional observation instrument was developed to code offensive behavioral factors during clutch moments. Detailed elements of each offensive possession were systematically observed to capture characteristics that may influence offensive outcomes in clutch moments.

Previous studies have applied inconsistent definitions of the “clutch” period, particularly in end-game situations, with commonly used score-differential thresholds including ≤ 3, ≤ 5, or ≤ 8 points, and no broad consensus has been established [13, 22, 23]. To enhance comparability, the present study follows the commonly used NBA criterion (the final 5 minutes with a score difference of ≤ 5 points) and adapts it to the FIBA competition format (10-minute quarters) by redefining the critical time window as the final 3 minutes of regulation play and all overtime periods [12]. This more conservative time setting was intended to more precisely capture phases of highest decision-making pressure and tactical concentration in FIBA games, thereby improving contextual consistency and criticality. In addition, to ensure that the selected possessions reflect genuinely close-game situations, only games with a final score difference of ≤ 5 points were included. Accordingly, the clutch period in this study was defined as all offensive possessions occurring during the final 3 minutes of regulation and all overtime periods in games with a final score difference of ≤ 5 points.

We first downloaded the full game videos from the two most recent FIBA Men’s Basketball World Cups (182 games in total) and identified 41 games that met the final-margin criterion (≤ 5 points). After video clipping and possession-by-possession coding, 727 offensive possessions were initially collected. Of these, 172 possessions were excluded due to possessions stopped by intentional fouls committed by the defense (n = 68), possessions in which the offense failed to reach a set formation or committed a turnover before a recognizable play-types could be established (n = 94), and rushed backcourt shot attempts due to insufficient remaining time (n = 10). The remaining dataset comprised 555 offensive sequences; among them, 39 sequences included two consecutive offensive opportunities, five included three consecutive opportunities, and one sequence included four consecutive opportunities. Following prior methodology, all sequences involving consecutive offensive rebounds were treated as a single offensive possession [10]; consequently, a total of 607 offensive possessions were included in the final analyses. All analyzed games were conducted in public competitive settings, and video footage was retrieved from the official FIBA Game Videos platform (gamevideos.fiba.com). The dataset was fully anonymized and contained no personal or identifiable information; therefore, formal ethical approval was not required.

### Instruments

After defining the research questions, an observation instrument tailored to the objectives of the present study was developed. The construction of this instrument was based on: (i) established theoretical frameworks; (ii) empirically validated criteria and categories from previous observational studies [8, 10, 16, 17]; and (iii) additional indicators specifically introduced to accommodate the context of this research. The development process proceeded as follows. First, an analysis panel was formed consisting of three researchers holding doctoral degrees, each with more than 10 years of experience in observational methodology and basketball performance analysis. Next, relevant conceptual definitions and prior literature were systematically reviewed to clarify the research aims and to preliminarily determine the initial set of observational indicators and coding rules. On this basis, pilot coding was conducted on a subset of the sample to assess the feasibility and operability of the instrument. Subsequently, the research team held multiple consensus meetings to evaluate and refine the instrument in terms of conceptual validity, category boundaries, and coding consistency. After three iterative rounds of discussion and testing, the final observation instrument was established. As shown in Table 1 and Figure 1, the instrument comprises three dimensions, contextual constraints, tactical choices, and finishing quality, encompassing a total of 10 indicators and 47 categories, among which the definition of play-types was adapted from the Synergy Sports classification system [17]. The instrument was designed according to the principles of exhaustiveness and mutual exclusivity.

**TABLE 1:** Classification of the play-type and tactical scenarios for the ball possession analyses.

Criteria	Code	Description
Score-line margin at the start of the ball possession	Tie Leading 1–3 Leading 4–6 Leading 6+ Behind 1–3 Behind 4–6 Behind 6+	The play begins with a tied score The play begins with a leading 1–3 score The play begins with a leading 4–6 score The play begins with a leading 6+ score The play begins with a behind 1–3 score The play begins with a behind 4–6 score The play begins with a behind 6+ score

Ball possession starting scenario	Ball in play (BP) Offensive sideline throw-in (OST) Offensive baseline throw-in (OBT) Defensive baseline throw-in (DBT) Defensive sideline throw-in (DST) Offensive rebound (OR)	The play begins with a live ball The play begins with a sideline throw-in in the frontcourt The play begins with a baseline throw-in in the frontcourt The play begins with a baseline throw-in in the backcourt The play begins with a sideline throw-in in the backcourt The play begins with an offensive rebound

Passes used	Pass 0 Pass 1 Pass 2 Pass 3 Pass 4 Passes ≥ 5	Zero passes are made during the play One pass is made during the play Two passes are made during the play Three passes are made during the play Four passes are made during the play Five or more passes were made during the possession

Ending court zone	Zone 1 Zone 2 Zone 3 Zone 4 Zone 5 Zone 6 Zone 7 Zone 8	The play ends in the paint The play ends in the right side mid-range by the basket The play ends in the above-the-free-throw-line two-point area The play ends in the left side mid-range by the basket The shot ends in the left corner behind the three-point line The shot ends in the left wing behind the three-point line The shot ends in the top of the key behind the three-point line The shot ends in the right wing behind the three-point line

	Zone 9	The shot ends in the right corner behind the three-point line

Possession duration	Time 0–8 s Time 9–16 s Time 17–24 s	The team ends the play with between 0 and 8 s of possession remaining The team ends the play with between 9 and 16 s of possession remaining The team ends the play with between 17 and 24 s of possession remaining

Ending player position	Guard Forward Center	The finisher’s position was obtained from the official FIBA website. The possession ended with a guard, forward, or center.

Possession outcome	Basket	The play ends with a basket

	No basket	The play ends without a basket

Play-type	Spot Up	Possession ending event finished with a catch and shot or catch and drive. They can be catching and shooting, attacking a close out by dribbling into a pull-up, dribbling into a floater, or driving to the rim

	P&R Ball Handler	Possession ending event finished by the ball-handler in the pick-and-roll. Includes off dribble shots or direct shots before dribbling off the screen, pull-ups and floaters, by that player. Includes also possessions where the ball handler rejects the screen (dribbles away from the screen)

	Transition	Quick offense, resulting in scoring or taking the shot before the defense is set

	Post Up	Playing 1 on 1 near to the basket with back turned to the basket (in most cases this is played by Centers)

	Cut	Possession ending event where the player without a screen cuts out or toward the ball to receive it. Includes backdoor cuts and flash cuts and spacing when a player is getting open near the basket

	Isolation	Possession ending event where the player is in 1-vs-1 situation trying to size up and create space from his defender

	P&R Roll Man	Possession ending event where the screener receives the ball. These are the slips, rolls, and pops from screeners in the pick-and-roll

	Off Screen	Possession ending event generated by a player running off a screen, whether it be a pin-down, flare screen, elevator screens, or any other of the plethora of screen variations before they receive the ball. This includes curls and fades

	Put Backs	Possession ending event where a tip in or quick shot happens after offensive rebound

	Hand Off	Possession ending event where the screener with or without dribbling hands over the ball to a player cutting towards him, acting as an immediate screener creating space for his teammate

After timeout	YES	The play begins after the timeout

	NO	The play does not begin after the timeout

**FIG. 1:**
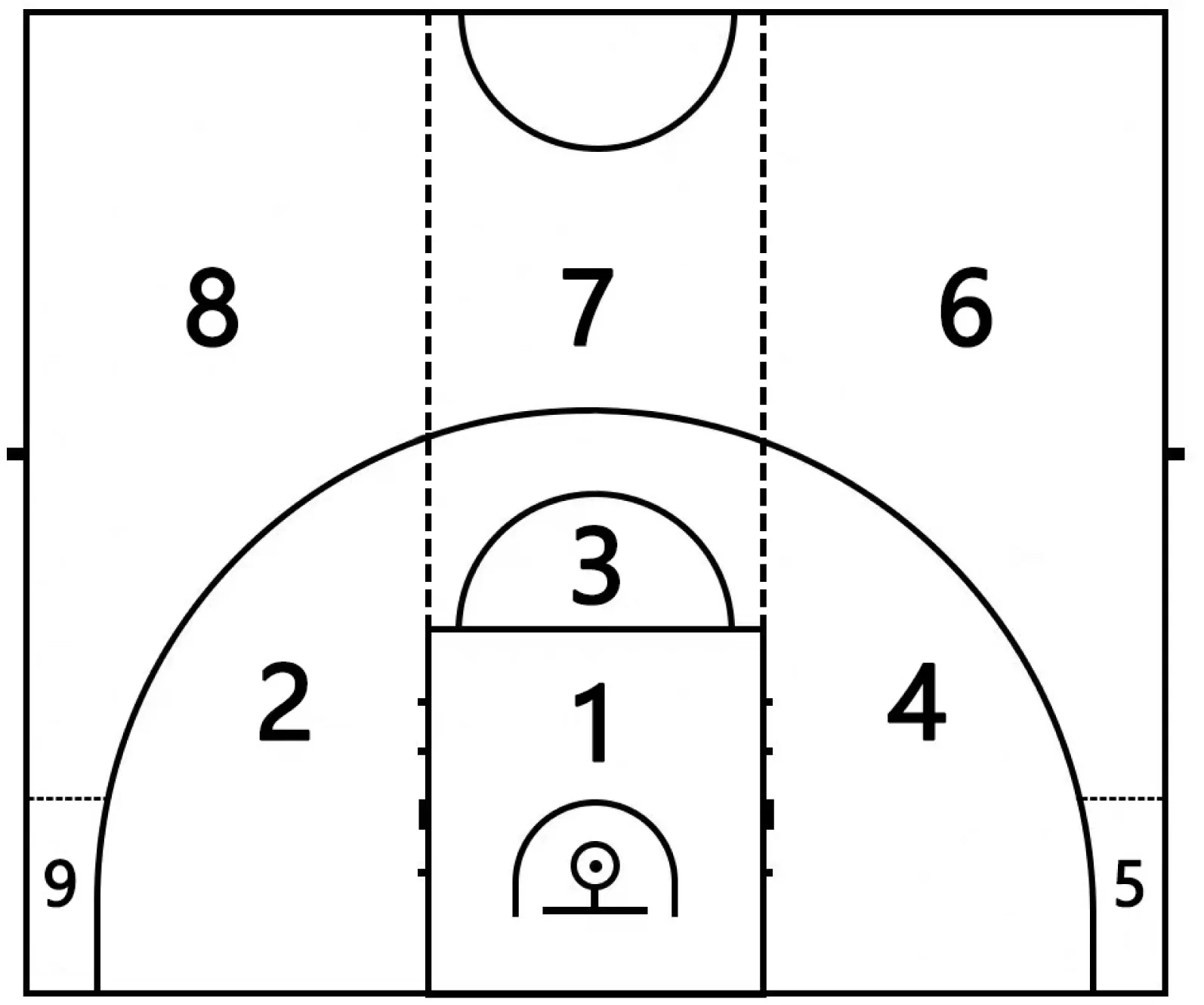
Classification of ending court zones used in the observational instrument. Note: Zone 3 is defined as all two-point field goals attempted above the free-throw line, excluding shots taken from Zones 1, 2, and 4.

### Procedure and reliability

All game videos were downloaded from the official website of the International Basketball Federation (https://gamevideos.fiba.com/). After downloading and filtering the games according to the inclusion criteria, the final three minutes of the fourth quarter and all overtime periods from each game were extracted, merged into a single video file, and chronologically organized and labeled. Video editing and processing were conducted using Hudl Sportscode (v 11, Agile Sports Technologies Inc., NB, USA). Based on the observational instrument developed for this study (Table 1 and Figure 1), two basketball performance analysts, each with more than five years of professional video-analysis experience, independently coded all game segments to ensure accuracy and consistency in data annotation. All offensive actions were recorded according to the operational standards of the observational tool, and the system automatically generated the corresponding Excel data files. To minimize intra-observer variability and ensure consistent coding, the observer participated in ten systematic training sessions before the formal coding process. During training, a set of 100 possessions, excluded from the final dataset, was used for practice observation and coding. Coding was performed only when the observer’s judgments were fully consistent with all classification criteria defined in the observational instrument. Four weeks after the initial data analysis, a random subset of 122 possessions (approximately 20% of the final sample) was recoded to assess both intra- and inter-observer reliability. Cohen’s Kappa coefficients were calculated and demonstrated excellent reliability across all indicators, with “play-types” showing the lowest, but still very high, reliability (intra-observer = 0.97, inter-observer = 0.96). Following the criteria proposed by Fleiss et al. [24], all variables achieved “almost perfect” levels of agreement (Table 2).

**TABLE 2:** Intra and inter-observer agreement.

Criteria	Kappa coefficient	

	Intra-observer	Inter-observer
Ball possession starting scenario	1.00	1.00
Score-line margin at the start of the ball possession	0.98	0.98
Ending court zone	1.00	1.00
Passes used	1.00	1.00
Possession duration	0.99	0.99
Ending player position	1.00	1.00
Play-type	0.97	0.96
After Timeout	1.00	1.00
Possession outcome	1.00	1.00

### Statistical analysis

First, to identify significant associations between the research variables and the binary outcome variable “scoring success,” chi-square tests of independence were conducted to examine statistically significant relationships between the studied criteria. To support the reliability of the chi-square approximation, the minimum expected cell count and the percentage of cells with expected counts below 5 were extracted from the test output and reported. Cramér’s V was calculated to evaluate effect size (ES), with thresholds set at p < 0.05 for statistical significance and 0.10 (small), 0.30 (medium), and 0.50 (large) for ES magnitudes [25]. When a significant chi-square value was observed, post-hoc analyses were conducted by examining adjusted standardized residuals (ASR) to identify the specific cells contributing to the overall association, with |ASR| ≥ 1.96 indicating a statistically meaningful deviation from the expected frequency (two-tailed p < 0.05).

Next, to more flexibly and robustly handle high-dimensional data and potential multicollinearity, thereby enhancing the generalizability of the findings, we evaluated a range of widely used machine-learning classifiers spanning different methodological categories, including tree-based models (Decision Tree, Random Forest), boosting algorithms (XGBoost, LightGBM, CatBoost), linear models (Logistic Regression, Ridge Regression, Linear SVM), instance-based learning (K-Nearest Neighbors), and neural networks (Multilayer Perceptron classifier) [26]. Based on overall predictive performance and stability across validation folds, the best-performing model was selected for subsequent interpretability analysis. Specifically, for all models, the dependent variable was recoded into a binary outcome, defined as “success” (made field goals and made free throws following fouls) and “non-success” (missed shots, turnovers, and blocked shots). This recoding was motivated by several considerations. First, it is consistent with the core evaluative objective of basketball offensive possessions, namely, distinguishing whether each possession ultimately results in scoring, thereby providing a more direct reflection of offensive efficiency. Second, the use of binary classification helps reduce issues related to class imbalance and sample sparsity, consequently improving model stability and predictive performance. The following variables were included as predictors (Table 1): scoreline marginal, the start of the possession, ball possession starting scenario, passes used, play-types, possession duration, ending zone, ending player position, and timeout. Model performance was evaluated using accuracy, precision, recall, F1-score, and the area under the receiver operating characteristic curve (AUC) [27]. The dataset was randomly split into training (80%) and testing (20%) sets, and hyperparameter tuning was conducted on the training data using stratified 10-fold cross-validation with a fixed random seed (42) to enhance model robustness and generalizability. RandomizedSearch-CV was employed to optimize hyperparameters efficiently while reducing computational burden [28].

To interpret the complex machine-learning models, SHAP values were computed to quantify the contribution of each feature to the predicted probability of scoring for each possession. SHAP provides additive, locally accurate attributions with consistent global aggregation [28], enabling us to (i) rank influential predictors at the global level; and (ii) identify the direction and potential non-linear effects of key variables. This approach enhances the practical interpretability of the models and supports the translation of model outputs into coaching-relevant decision cues.

## RESULTS

Table 3 presents the results of the chi-square tests of independence between the dependent variable “scoring success” and all examined predictors. Significant associations were observed for play-types (p < 0.001), possession duration (p = 0.009), and ending zone (p = 0.001), whereas no statistically significant relationships were found for the remaining variables (p > 0.05). The corresponding effect sizes for each variable are also reported in Table 3. Post-hoc analyses based on adjusted standardized residuals (ASR) further clarified the sources of these associations. For the ending zone, shots taken from zone 1 resulted in a significantly higher-than-expected frequency of scoring success (ASR = 6.1), whereas zones 6 and 8 showed a significantly higher-than-expected frequency of unsuccessful outcomes (ASR = 3.7 and 2.8, respectively). With respect to play-types, put-back actions and transition plays were associated with a significantly higher-than-expected frequency of scoring success (ASR = 3.2 and 2.7, respectively), while isolation plays and spot-up plays exhibited a significantly higher-than-expected frequency of missed shots (ASR = 2.3 and 2.9, respectively). Regarding possession duration, possessions lasting 1–8 s were characterized by a significantly higher-than-expected frequency of scoring success (ASR = 3.1), whereas possessions lasting 9–16 s showed a significantly higher-than-expected frequency of unsuccessful outcomes (ASR = 2.2).

**TABLE 3:** Results of Independent Chi-Square Test

	Dimension	Basket (n = 310, 51.1%)	No Basket (n = 297, 48.9%)	Min Exp.	Cells Exp. < 5, n (%)	χ^2^	ES	P
Score-line margin at the start of the ball possession	Tie	42 (13.5%)	41 (13.8%)	8.81	0.0%	6.227	0.101	0.398
	Leading 1–3	56 (18.1%)	60 (20.2%)					
	Leading 4–6	25 (8.1%)	32 (10.8%)					
	Leading 6+	7 (2.3%)	11 (3.7%)					
	Behind 1–3	86 (27.7%)	86 (29.0%)					
	Behind 4–6	64 (20.6%)	47 (15.8%)					
	Behind 6+	30 (9.7%)	20 (6.7%)					

Ball possession starting scenario	Offensive rebound (OR)	42 (6.5%)	25 (8.4%)	11.25	0.0%	5.845	0.096	0.361
	Defensive baseline throw-in (DBT)	124 (40.0%)	118 (39.7%)					
	Defensive sideline throw-in (DST)	20 (6.5%)	23 (7.7%)					
	Ball in play (BP)	79 (25.5%)	72 (24.2%)					
	Offensive sideline throw-in (OST)	37 (11.9%)	44 (14.8%)					
	Offensive baseline throw-in (OBT)	8 (2.6%)	15 (5.1%)					

Passes used	Pass 0	85 (27.4%)	68 (22.9%)	18.59	0.0%	7.765	0.113	0.170
	Pass 1	79 (25.5%)	93 (31.3%)					
	Pass 2	71 (22.9%)	60 (20.2%)					
	Pass 3	33 (10.6%)	36 (12.1%)					
	Pass 4	27 (8.7%)	17 (5.7%)					
	Passes ≥ 5	15 (4.8%)	23 (7.7%)					

Play-type	Cut	17 (5.5%)	12 (4.0%)	8.81	0.0%	31.783	0.229	**< 0.001**
	Hand Off	7 (2.3%)	11 (3.7%)					
	Isolation	10 (3.2%)	22 (7.4%)					
	Off Screen	25 (8.1%)	30 (10.1%)					
	P&R Ball Handler	74 (23.9%)	71 (23.9%)					
	P&R Roll Man	30 (9.7%)	23 (7.7%)					
	Post Up	19 (6.1%)	14 (4.7%)					
	Put Backs	20 (6.5%)	4 (1.3%)					
	Spot Up	54 (17.4%)	81 (27.3%)					
	Transition	54 (17.4%)	29 (9.8%)					

Possession duration	Time 0–8 s	54 (17.4%)	29 (9.8%)	74.86	0.0%	9.493	0.125	**0.009**
	Time 9–16 s	135 (43.4%)	123 (38.7%)					
	Time 17–24 s	97 (31.2%)	79 (24.8%)					

Ending court zone	Zone 1	162 (52.3%)	90 (30.3%)	4.89	5.0%	50.331	0.228	**< 0.001**
	Zone 2	11 (3.5%)	11 (3.7%)					
	Zone 3	6 (1.9%)	4 (1.3%)					
	Zone 4	25 (8.1%)	23 (7.7%)					
	Zone 5	6 (1.9%)	14 (4.7%)					
	Zone 6	23 (7.4%)	51 (17.2%)					
	Zone 7	45 (14.5%)	44 (14.8%)					
	Zone 8	25 (8.1%)	45 (15.2%)					
	Zone 9	7 (2.3%)	15 (5.1%)					

Ending player position	Guard	168 (54.2%)	164 (55.2%)	41.59	0.0%	1.777	0.053	0.411
	Forward	90 (29.0%)	100 (33.7%)					
	Center	52 (16.8%)	33 (11.1%)					

After TimeOut (ATO)	NO	282 (91.0%)	267 (89.9%)	28.18	0.0%	0.200	0.018	0.654
	Yes	28 (9.0%)	30 (10.1%)					

Significant associations (p < 0.05) are bolded

Table 4 summarizes the performance metrics of all evaluated models on the test set. Overall, the Random Forest demonstrated the best or near-best performance across all metrics (Accuracy = 0.6803, Precision = 0.6810, Recall = 0.6803, F1-score = 0.6803, ROC AUC = 0.7070) and was therefore selected as the final model for subsequent interpretability analyses. By comparison, XGBoost, Logistic Regression, and LightGBM formed a second performance tier (Accuracy ≈ 0.656, ROC AUC ≈ 0.687–0.692), whereas linear models (Linear SVM, Ridge Regression) and instance-based or neural network approaches (KNN, MLPClassifier) showed relatively weaker performance. The single Decision Tree yielded the poorest predictive results (Accuracy = 0.5738, ROC AUC = 0.5930), indicating pronounced overfitting and limited generalization ability. For the Random Forest, the optimal hyperparameter configuration identified via stratified 10-fold cross-validation was max_depth = 60, min_samples_split = 5, min_samples_leaf = 4, and max_features = log2. This configuration reflects a reasonable trade-off between model complexity and generalization: increased tree depth enhances the modeling of non-linear relationships, while split and leaf constraints together with feature subsampling effectively mitigate overfitting, thereby improving the stability and discriminative capacity of the model in predicting offensive outcomes during clutch possessions.

**TABLE 4:** Performance metrics for the ten models implemented

	Accuracy	Precision	Recall	F1-score	AUC
Random Forest	0.6803	0.6810	0.6803	0.6803	0.7070
XGBoost	w0.6557	0.6560	0.6557	0.6552	0.6917
Logistic Regression	0.6557	0.6560	0.6557	0.6552	0.6866
Linear SVM	0.6475	0.6475	0.6475	0.6475	0.6890
Ridge Regression	0.6311	0.6311	0.6311	0.6311	0.6634
LightGBM	0.6557	0.6560	0.6557	0.6552	0.6683
KNN	0.6311	0.6344	0.6311	0.6276	0.6313
CatBoost	0.6148	0.6160	0.6148	0.6144	0.6476
ANN (MLPClassifier)	0.6066	0.6070	0.6066	0.6053	0.6653
Decision Tree	0.5738	0.5795	0.5738	0.5689	0.5930

Note: AUC = area under the curve, KNN = K-Nearest Neighbors

Figures 2–3 present the SHAP value distributions and global importance ranking for the Random Forest model. Ending-zone variables were the strongest predictors of scoring success; in particular, ending in 1 was associated with predominantly positive SHAP values, indicating a higher predicted scoring probability. Possession duration was the next most influential factor, with shorter possessions (1–8 s) contributing positively and longer possessions (9–16 s) contributing negatively. Play-types ranked third, with spot-up and isolation both tending to reduce the probability of scoring. Score difference and ending player position had comparatively smaller effects on scoring success. The SHAP results of XGBoost and Logistic Regression, which ranked in the second tier of overall model performance, are presented in Supplementary Figs. 1–4. The results showed a high degree of consistency among the SHAP values of XGBoost, Logistic Regression, and the Random Forest model, further highlighting the robustness and reliability of the study finding.

**FIG. 2:**
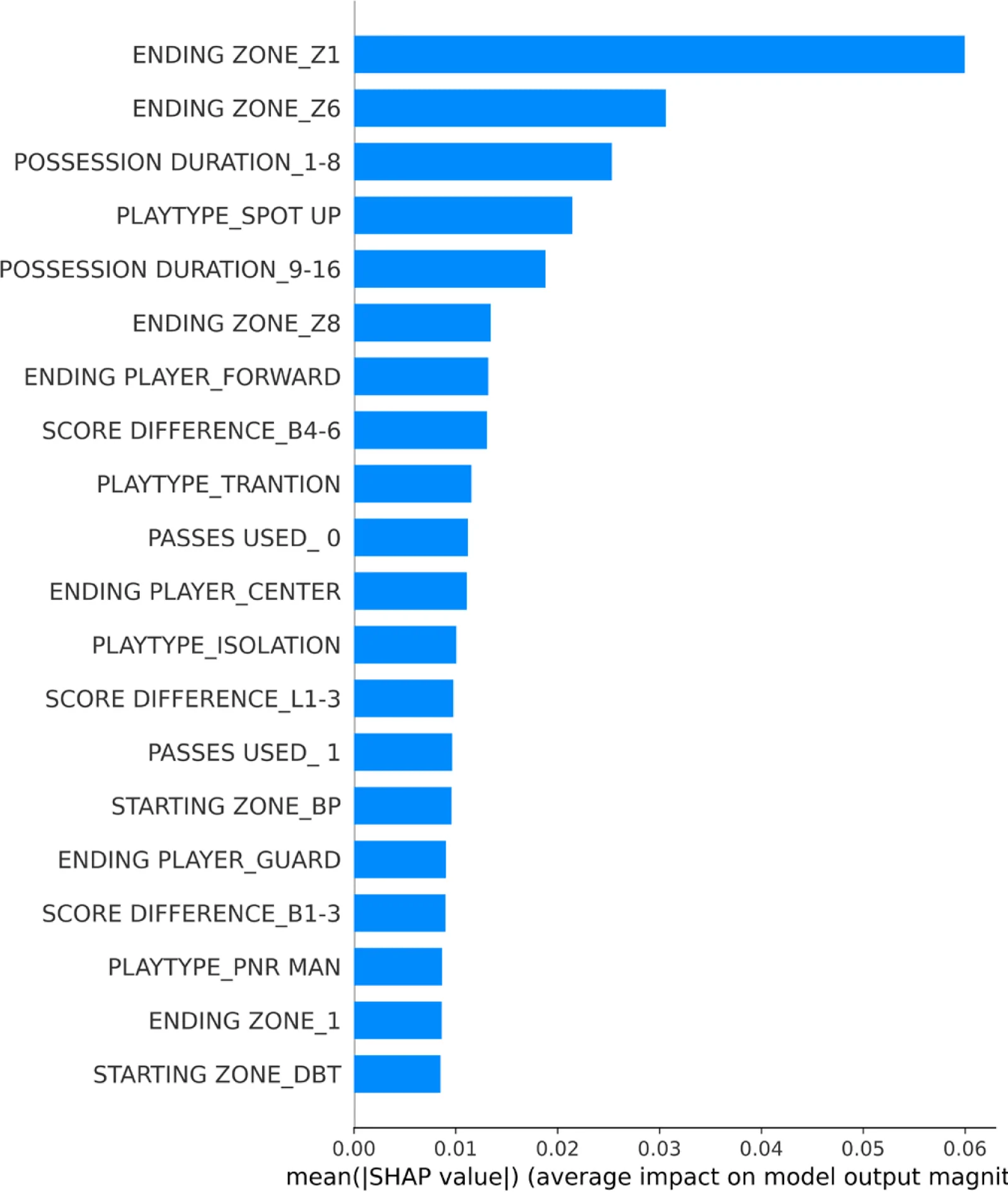
Global feature importance ranking based on mean absolute SHAP values for the Random Forest model. Note: Bars represent the mean absolute SHAP value (mean(|SHAP|)) of each predictor across all possessions, indicating its average contribution to the model output magnitude. Features are ranked in descending order of importance.

**FIG. 3:**
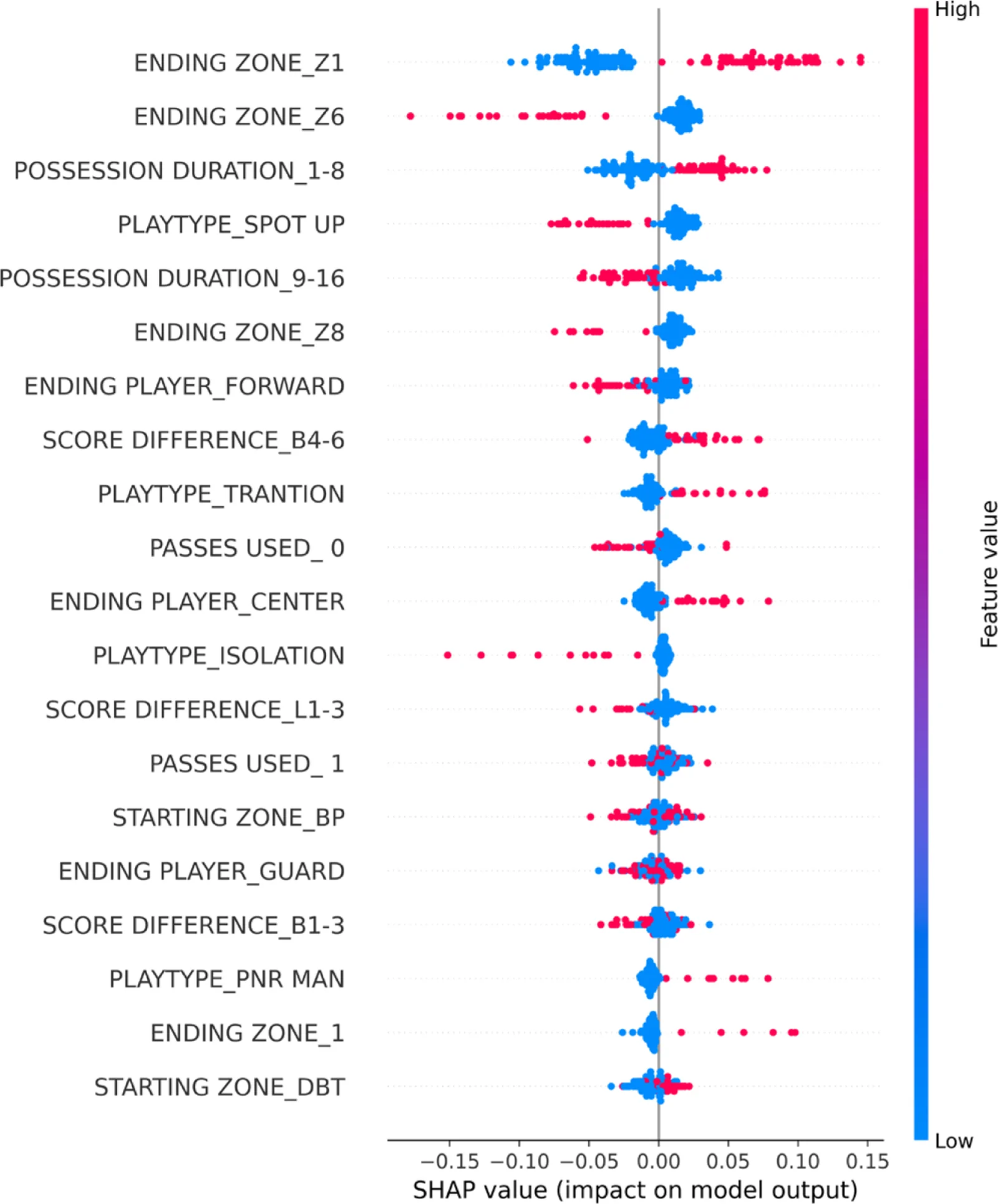
Global feature importance ranking based on mean absolute SHAP values for the Random Forest model. Note: Features are ranked by mean absolute SHAP value (y-axis). Each dot represents one possession, with color indicating the feature value (low = blue, high = red). The x-axis shows SHAP values, where positive values increase and negative values decrease the model-predicted probability of scoring.

## DISCUSSION

This study aimed to evaluate the key factors influencing offensive success during clutch moments in elite international men’s basketball. Ten different types of machine learning models were systematically compared to identify the best-performing predictive model, and SHAP analysis was subsequently applied to interpret the contribution and directional effects of KPIs on the model outputs.

Among the 607 offensive possessions included in the analysis, 310 (51.1%) resulted in scoring success, whereas 297 (48.9%) did not. Under the clutch-time conditions defined in the present study, offensive success exhibited a relatively balanced distribution, which is consistent with findings from previous research conducted in both NBA game contexts and international competitions such as the European Basketball Championships [8, 10].

More importantly, the present findings suggest the potential existence of interaction effects among tactical and contextual variables during clutch moments. In other words, offensive success may not depend on isolated tactical variables, but rather on an integrated possession pattern characterized by rapid offensive execution and coordinated team-oriented actions. Specifically, shorter possessions combined with dynamic collective play-types, such as transition offense and pick-and-roll actions, appeared to create more favorable scoring opportunities. In contrast, prolonged isolation possessions may further increase defensive pressure and reduce finishing quality. Moreover, transition and pick-and-roll actions were more likely to generate successful interior finishes, while centers demonstrated a greater likelihood of successful finishes arising from pick-and-roll situations. Collectively, these findings support the proposed pathway linking contextual constraints, tactical choices, and finishing quality during clutch moments [10].

From a theoretical interaction perspective between play-types and possession duration, transition offense and pick-and-roll actions executed within 1–8 s were positively associated with scoring success, whereas isolation and spot-up shooting occurring within 9–16 s demonstrated relatively low efficiency. These findings are consistent with previous studies on clutch moments in the EuroLeague, which likewise reported the relatively low efficiency of isolation and spot-up shooting (0.78–0.98 points per possession) [16]. Similarly, Christmann et al. [10] emphasized that team-oriented offensive actions tend to be more effective than individual ball-dominant plays in NBA games. A plausible mechanism underlying these results is that isolation reduces tactical diversity and increases offensive predictability, enabling defenses to anticipate and intervene more effectively. In addition, the negative effect of spot-up shooting may stem from the greater level of shooting consistency required for long-range attempts during clutch moments, which may become more difficult to maintain under accumulated fatigue and psychological pressure [29]. In contrast, screening and pick-and-roll actions can generate spatial and numerical advantages through mismatches, delays, and defensive rotations, with possessions finished by the screener often yielding higher efficiency [30]. Likewise, transition offense exploits superior spacing and numerical advantages before the defense is fully organized, and has previously been identified as one of the most effective offensive strategies in basketball [31]. Overall, these findings suggest that teams may benefit during clutch moments from selectively increasing offensive tempo, emphasizing early decision-making, and rapid finishing. Coaches should therefore maximize the use of screening and pick-and-roll actions to enhance finishing quality, while using isolation more cautiously to improve scoring success in critical possessions.

From a theoretical interaction perspective between play-types and the ending zone, transition offense and pick-and-roll actions demonstrated higher scoring success rates when possessions were finished in the restricted area. In contrast, spot-up shooting showed lower scoring efficiency when finished from the three-point line. However, shot-selection tendencies vary across different score-related game contexts and coaching tactical styles. For teams in a leading position, attacking the interior not only enhances scoring stability but also consumes time, thereby maximizing the likelihood of maintaining the lead. Conversely, although three-point attempts involve greater risk for trailing teams, their higher scoring value provides greater potential to reduce or overturn deficits within a short period. Indeed, previous research has identified three-point shooting performance as one of the key determinants of game outcomes [32]. Accordingly, teams that are behind often increase the proportion of three-point attempts during clutch moments to maximize comeback probability.

From a theoretical interaction perspective between play-types and finishing player position, possessions arising from pick-and-roll actions may show a higher scoring success rate when finished by centers rolling to the basket. This pattern is consistent with previous clutch-time findings based on samples from the Men’s European Basketball Championship [8]. From a mechanistic perspective, the absence of a defensive three-second rule in the FIBA regulatory framework allows defenses to maintain prolonged rim protection and establish more stable help-defense structures in the paint, thereby restricting penetration opportunities for guards and forwards and imposing greater demands on their finishing ability [33]. In contrast, centers are better equipped to cope with high-pressure defensive environments due to their physical advantages, ability to establish deep position, and proficiency in contact finishing near the basket. In conjunction with the present findings, this further underscores the tactical value of centers during clutch moments, suggesting that coaches may benefit from increasing the involvement of centers as finishers through targeted actions such as roll finishes, deep seals, and interior finishes following weak-side cuts, thereby improving scoring success in critical possessions.

Regarding the score-differential indicator, the results of this study revealed that possession efficiency increased for teams that were trailing, with the strongest effect observed when the deficit ranged from 4 to 6 points. This phenomenon reflects the inherent tension of close games: when the margin is only 1–3 points, a single successful possession may be sufficient to tie or overturn the score, whereas a deficit of 4–6 points typically requires at least two consecutive successful possessions to shift the competitive balance [34]. Notably, trailing teams did not exhibit passive or disengaged behavior. Instead, the disadvantaged position often heightens psychological urgency and effort [35], stimulating stronger action motivation and prompting players to make more decisive and assertive offensive choices, thereby improving scoring efficiency. Existing research

supports this interpretation. When scores are close, teams that are behind tend to adopt more aggressive offensive strategies in an attempt to quickly equalize or regain the lead [36], whereas leading teams often focus on prolonging ball possession to reduce game time and preserve their advantage [37]. Moreover, findings from the European Men’s Basketball Championship revealed that trailing teams showed significantly higher scoring efficiency than leading teams during clutch moments [8]. This aligns with the present study and further underscores the psychological and tactical proactivity displayed by the trailing side in tightly contested scenarios. Collectively, these findings indicate that being at a score disadvantage can, to some extent, stimulate greater offensive intensity during clutch moments, thereby generating a brief yet pivotal counteracting force within the competition.

In basketball, timeouts provide coaches with crucial opportunities to adjust tactics, reorganize offensive and defensive structures while interrupting the momentum of opponents [38]. However, the findings of the present study indicated that timeouts did not demonstrate a statistically significant main effect on the probability of scoring on the first possession following a timeout during clutch moments in the Men’s Basketball World Cup. This may be because timeouts also provide defensive teams with opportunities to reorganize defensive strategies, implement targeted adjustments, and make personnel substitutions, which may partially or completely offset the potential offensive advantages associated with timeouts [18]. Collectively, the tactical effectiveness of timeouts during clutch moments may be constrained by psychological factors, information symmetry, and the dynamic state of the game, indicating that coaches should employ timeouts with caution in clutch moments.

This study compared ten different types of machine learning models to identify the most effective approach for predicting offensive success during clutch moments, with the results indicating that the Random Forest achieved the best overall performance. Unlike previous studies that primarily relied on basic statistical indicators (e.g., box score data) as predictive variables [39, 40], this study is the first to incorporate dynamic technical–tactical indicators into a predictive modeling framework. This methodological advancement extends the application of machine learning in basketball contextual analysis and highlights the potential value of integrating tactical variables into predictive systems. Moreover, the key variables identified through SHAP-based interpretability analysis were highly consistent with the results of traditional chi-square tests, further supporting the reliability and interpretability of SHAP in elucidating the internal decision mechanisms of “black-box” models. Although the overall predictive performance of the models was moderate, this outcome is reasonable given the inherent complexity of basketball offensive actions and the influence of numerous contextual factors [41]. Notably, the achieved performance was still clearly superior to random prediction, indicating meaningful practical applicability.

From a practical application perspective, the SHAP results provide an intuitive basis for identifying the key drivers of offensive success during clutch moments and can inform coaches and analysts in setting training priorities and tactical configurations for elite international competitions. Overall, clutch possessions should place greater emphasis on team-oriented actions and high-certainty finishing pathways. Specifically, increasing the involvement of centers as primary finishers, through low-post positioning following off-ball screens and roll finishes in pick-and-roll actions, may enhance shot quality, while excessive reliance on individual isolation plays should be reduced. Accordingly, training programs may incorporate scenario-based drills that simulate clutch conditions and emphasize different offensive approaches; for example, replicating late-game deficit situations in which a moderate increase in offensive tempo is encouraged to capitalize on early advantages and shift unfavorable game dynamics, thereby improving scoring success in critical possessions and overall winning probability.

This study has several limitations that need to be addressed. First, due to its observational design, potential confounding factors could not be strictly controlled, including team strength, game phase, ingame status, individual player ability, and defensive pressure; all of these factors may influence the development of possessions and ultimately affect offensive outcomes. Second, the offensive possession outcome was operationalized as a binary dependent variable, which may obscure differences in tactical value between various scoring types and non-scoring outcomes, thereby limiting a fine-grained interpretation of possession value and strategic impact. Third, although standardized coding procedures were applied, the coding of possessions and related indicators inevitably involved a certain degree of subjectivity. Finally, as the study focused on elite-level competitions, the generalizability of the findings is limited, and the conclusions may not be applicable to other competitive contexts or league systems.

Future research should expand the sample scope to include more seasons and different competitive systems (e.g., the NBA and top European leagues) to examine the robustness and generalizability of the model across varying playing styles and rule contexts. In addition, adopting multi-category outcome variables or continuous metrics based on expected points would allow for more fine-grained modeling of possession outcomes. Integrating richer data sources, such as defensive performance indicators and player spatiotemporal tracking data, would also provide a more comprehensive understanding of offense–defense interactions during clutch moments. Further work could evaluate the model’s performance in real-time or near-real-time settings and explore its integration with video-based decision support systems (DSS), thereby enhancing its practical utility for in-game analysis.

## CONCLUSIONS

This study explored offensive performance during clutch moments in elite international men’s basketball by integrating machine learning approaches, proposing an innovative analytical framework, and further extending the application of SHAP in basketball performance analysis. Through a systematic comparison of ten categories of machine learning models, the Random Forest was identified as the optimal model for predicting possession-level offensive outcomes in clutch moments. The results demonstrated that play-types, possession duration, and ending zone were all significantly associated with scoring outcomes during critical game phases, with ending zone exerting the strongest influence. SHAP analysis further revealed that “zone_1,” “possession duration_1–8 s,” and “ending player position_center” were positively associated with scoring success, whereas “ending zone_6,” “play-types_spot up,” and “possession duration_9–16 s” showed negative associations with scoring success. From a practical perspective, these findings may provide strategic implications for elite international competitions. Specifically, offensive organization during clutch possessions should emphasize team-oriented actions and high-certainty finishing pathways by increasing the involvement of centers as primary finishers, strengthening screening and pick-and-roll actions, and reducing excessive reliance on individual isolation plays to enhance offensive stability and scoring success in clutch moments.

## Supplementary Material

Analysis of key performance indicators influencing offensive effectiveness during clutch moments in elite international men’s basketball
