# Supplementary material for: Analysis of key performance indicators influencing offensive effectiveness during clutch moments in elite international men’s basketball
Source: Biol Sport. 2026 Jul 27;43:1663–75. doi: 10.5114/biolsport.2026.163367 (PMC13711642; doi:10.5114/biolsport.2026.163367)
Supplement: Supplementary file 1 [file JBS-43-58394-s1.pdf]

SUPPLEMENTARY MATERIALS

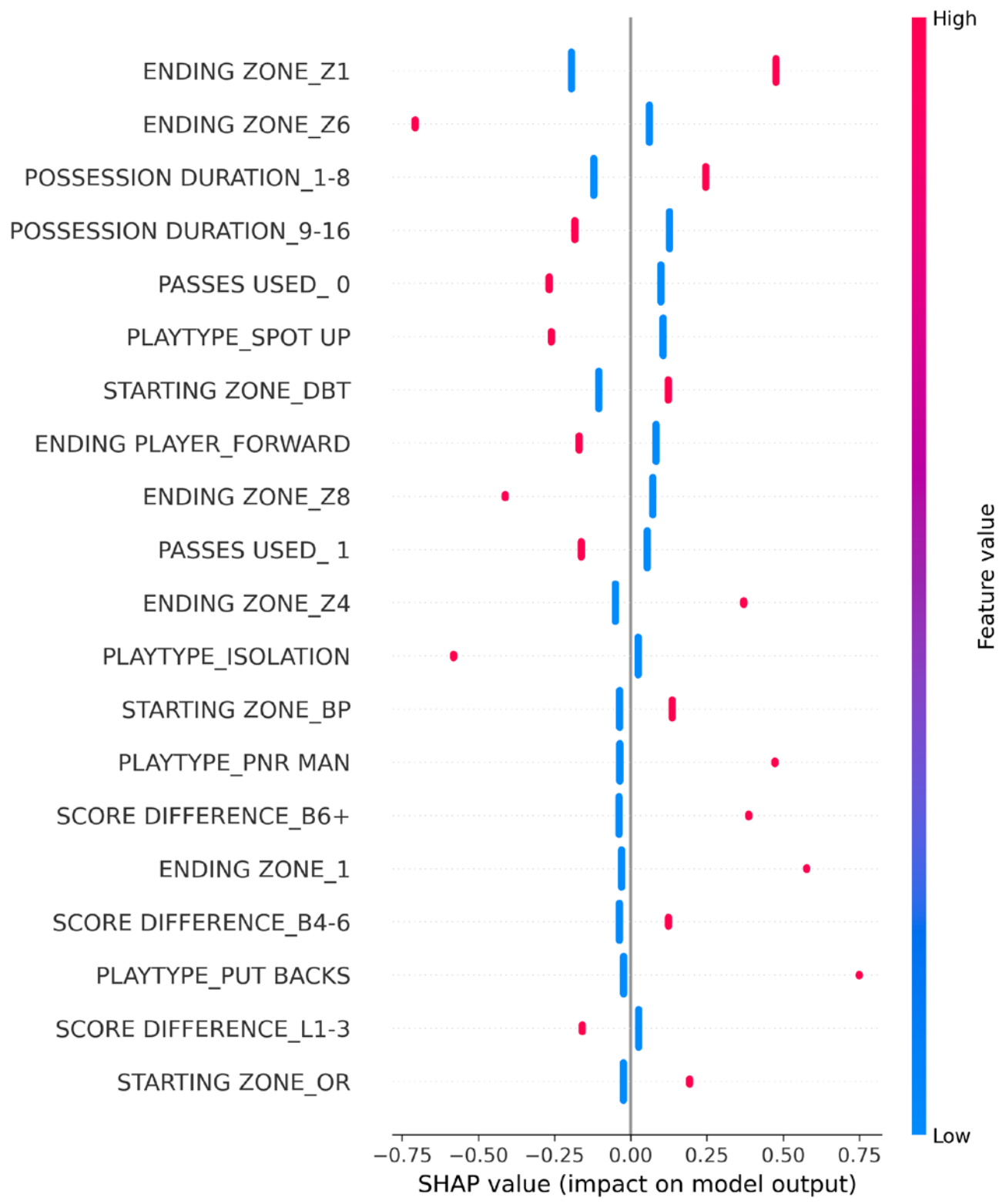

SUPPLEMENTARY FIG. 1. SHAP values of the Logistic Regression.

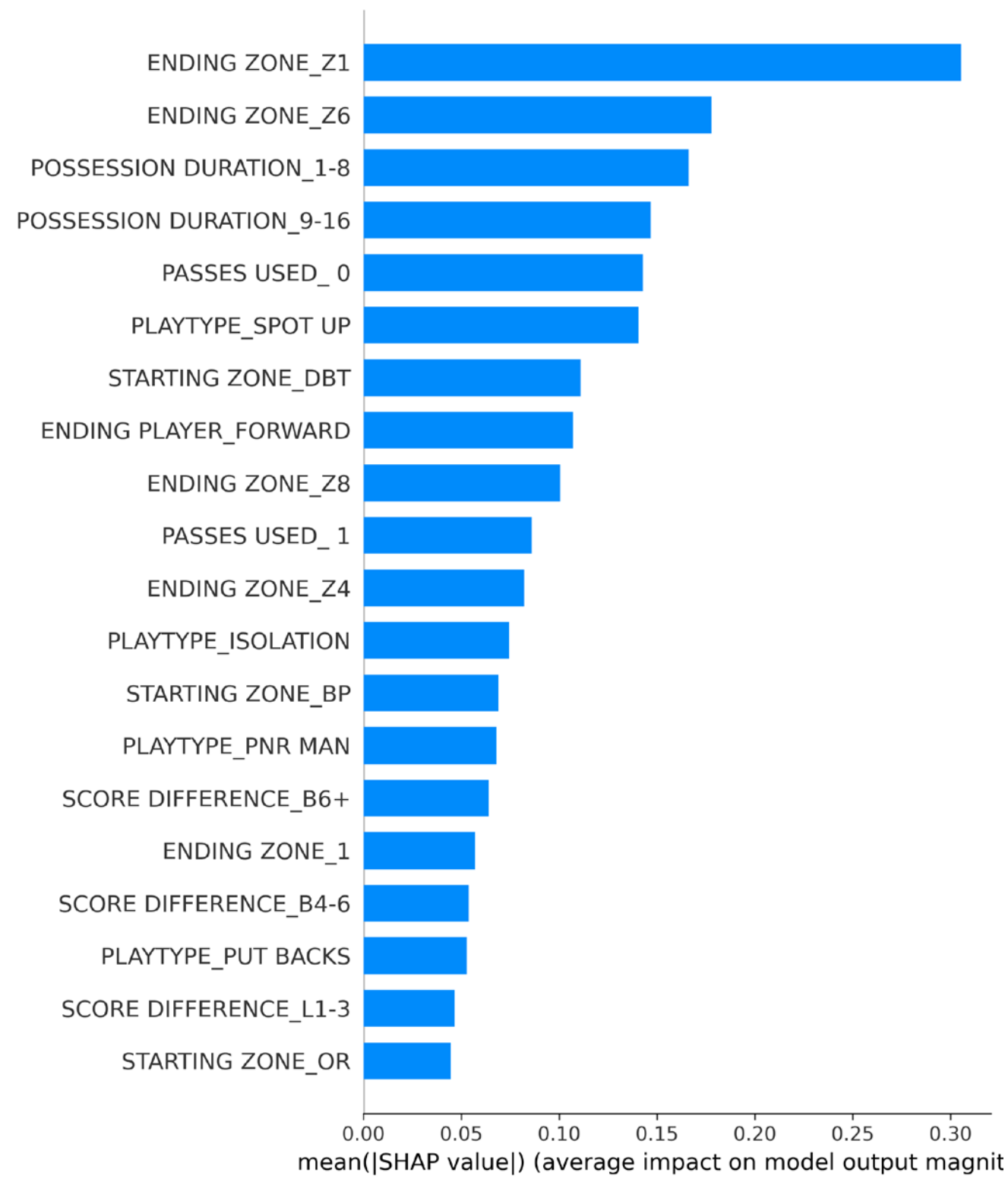

SUPPLEMENTARY FIG. 2. Feature-importance plots of Logistic Regression.
